# Acute Appendicitis in Young Children: A Persistent Diagnostic Challenge for Clinicians

**DOI:** 10.7759/cureus.2347

**Published:** 2018-03-19

**Authors:** Kewan A Hamid, Mohamed A Mohamed, Anas Salih

**Affiliations:** 1 Department of Combined Internal Medicine-Pediatrics, Hurley Medical Center, Michigan State University College of Human Medicine; 2 Michigan State University College of Human Medicine; 3 Department of Combined Internal Medicine-Pediatrics, Hurley Medical Center

**Keywords:** appendicular rupture, pediatric surgery, peritonitis, acute appendicitis

## Abstract

Acute appendicitis is a grave and life-threatening condition in children, accounting for one to two cases per 10,000 in children less than four years' old. Prompt diagnosis and management are imperative to prevent serious complications, such as abscess formation, perforation, bowel obstruction, peritonitis, and sepsis. In young children, however, the diagnosis of this condition is challenging. The delayed utilization of imaging may further delay the diagnosis due to concerns of exposure to ionizing radiation. Even with a prompt diagnosis, controversy persists regarding medical versus operative management in these young patients.

We report a case of a 21-month-old female who presented with fever, non-bilious, non-bloody emesis, and decreased tolerance for liquids and solids. The initial physical exam and imaging were suggestive of non-obstructive bowel distention. The patient was admitted to the pediatric floor. Overnight, the patient’s condition deteriorated severely and became septic. Repeat imaging revealed a 9-cm appendicular mass and a ruptured appendix. Antibiotic coverage was then broadened and the patient was transferred to the critical care unit for more intensive management. The patient’s septic condition improved over the upcoming few days and the parents elected to perform an elective appendectomy following resolution of the condition.

Atypical presentations are common in this population. The difficulty in obtaining a reliable history and physical examination findings makes the diagnosis even more challenging. Moreover, concerns with radiation exposure may delay the diagnosis and increase the risk of perforation and peritonitis. Thus, clinicians should have a high index of suspicion for acute appendicitis, particularly in young children, as this condition is commonly missed on initial presentation.

## Introduction

Acute appendicitis (AA) is a rare phenomenon in the pediatric population, accounting for one to two cases per 10,000 children under 4 years' old. Prompt diagnosis and management are imperative to prevent serious complications (such as abscess formation, perforation, and bowel obstruction) and life-threatening sequelae, such as peritonitis and sepsis [1]. In young children, however, the diagnosis of this condition is challenging. Their clinical presentations are heterogeneous, and it may be difficult to acquire reliable history and physical examination findings from a young historian [2-3]. Delayed utilization of imaging may further delay the diagnosis due to concerns with exposure to ionizing radiation. Even with a prompt diagnosis, controversy persists regarding medical versus operative management in these young patients [4]. Herein, we report a case of a 21-month-old female that presented with a fever.

## Case presentation

A 21-month-old female with no significant past medical or surgical history presented with a two-day history of intermittent fevers and vomiting. Parents reported non-bilious, non-bloody emesis prior to presentation. The patient also had decreased tolerance for liquids and refused all solids since the onset of symptoms. There was no diarrhea, melena, changes in bowel habits, rashes, or toxic ingestions reported. Parents denied sick contacts and recent travel history. Immunizations were up to date. The family and social history were noncontributory.

**Figure 1:**
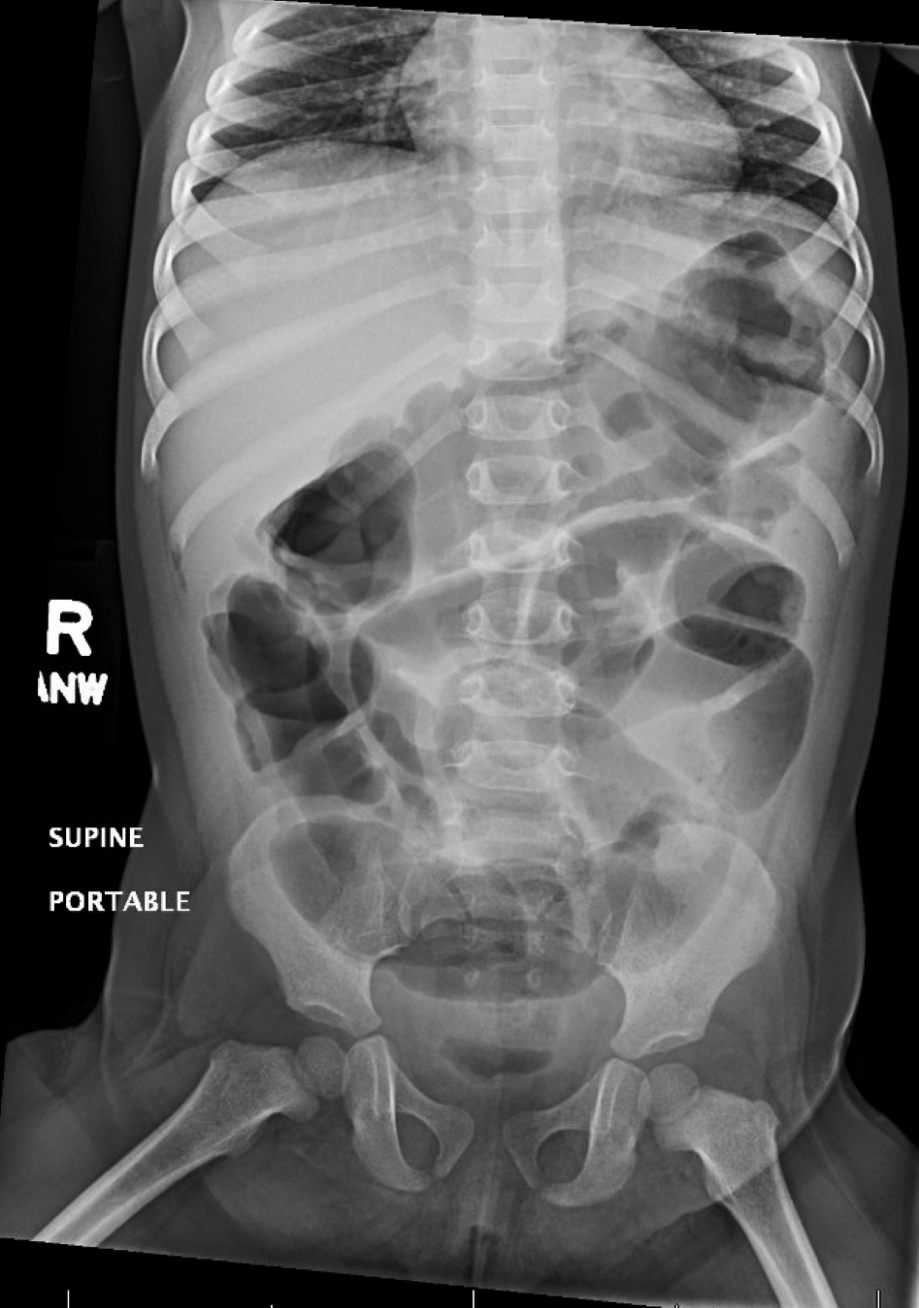
Initial radiograph on admission demonstrating non-obstructive gas patterns without evidence of any discrete masses or abnormal calcifications

**Figure 2:**
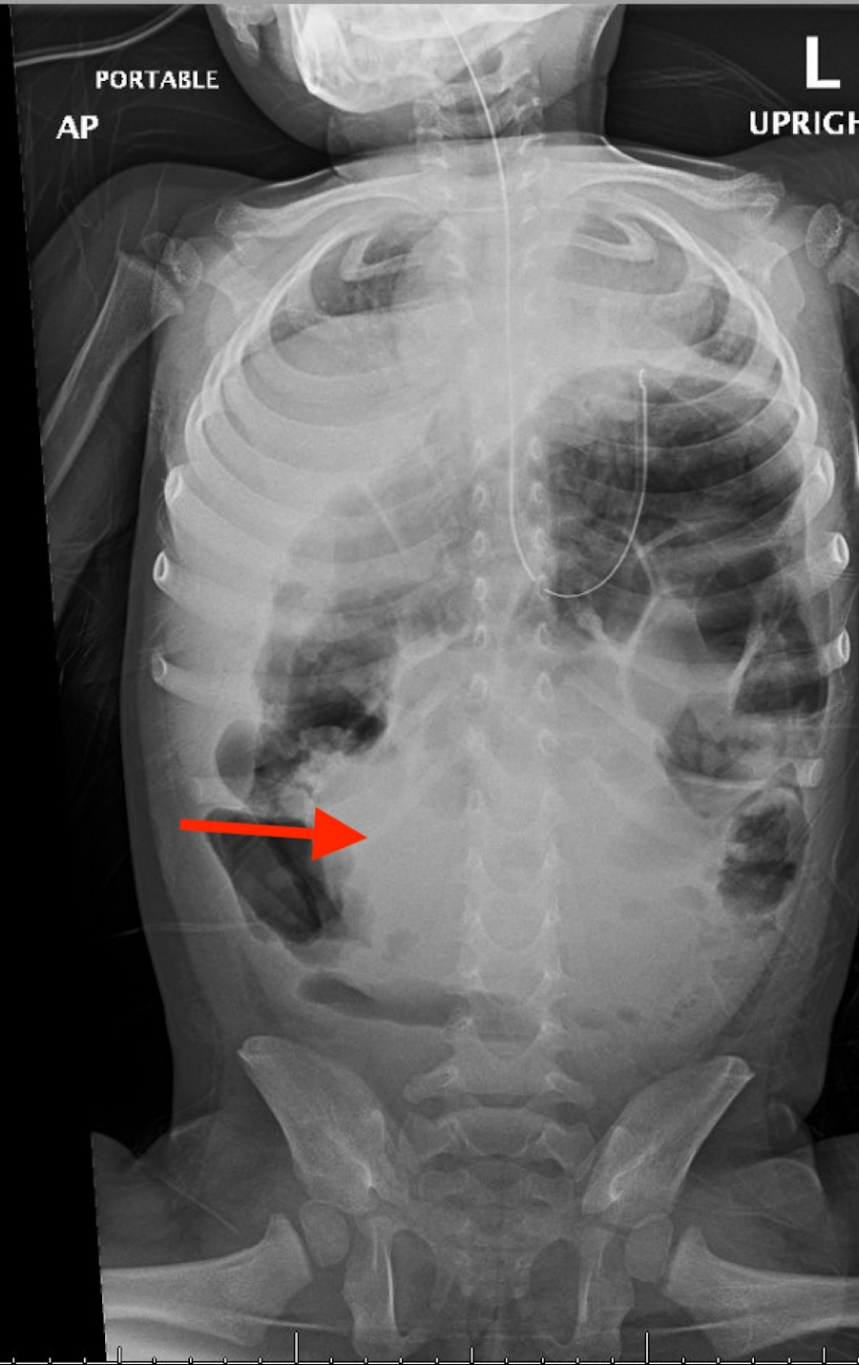
Repeat radiograph on day two of admission, demonstrating a midline soft tissue mass that is 9 cm in diameter

**Figure 3:**
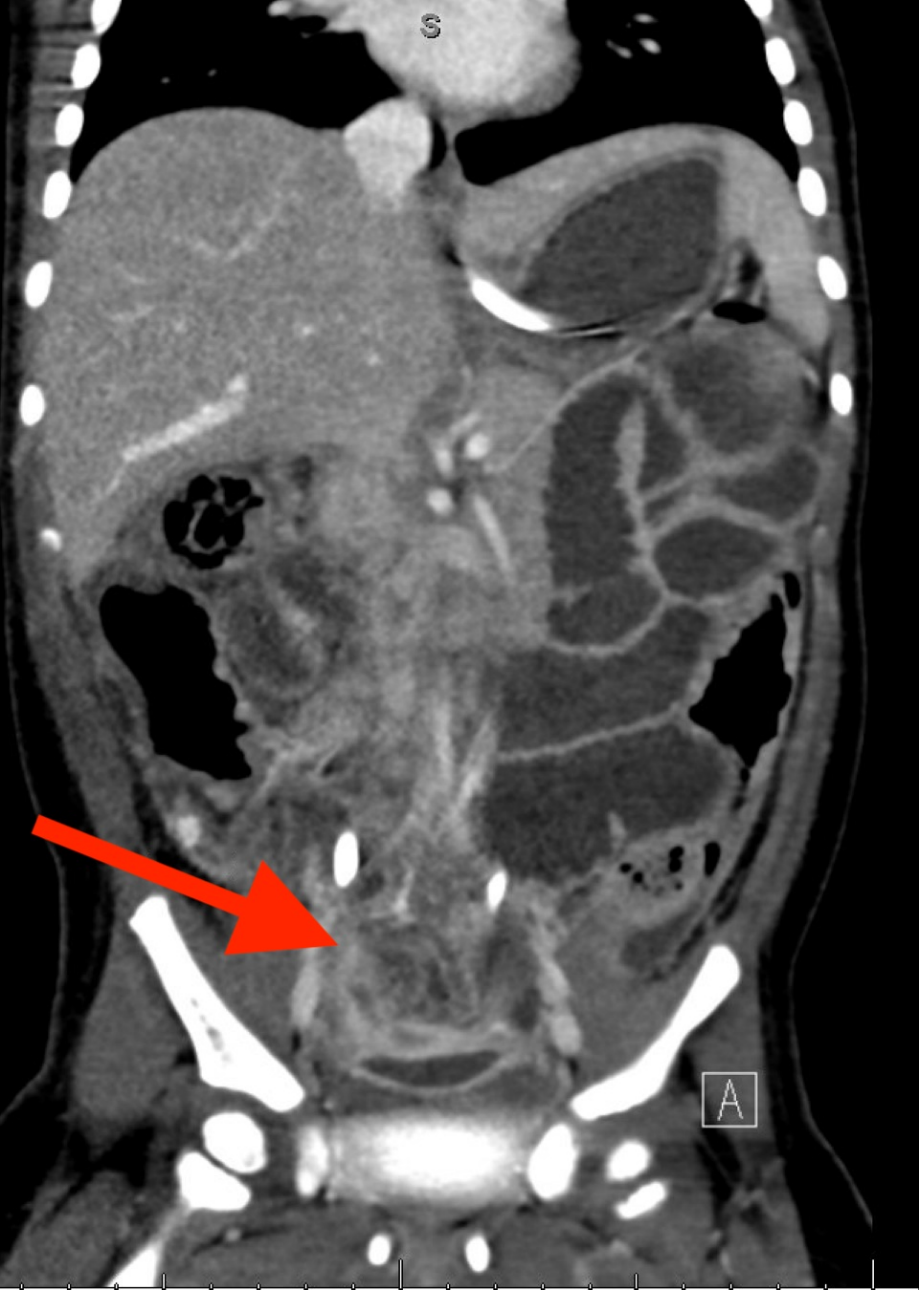
Computed tomography (frontal plane) on the second day of admission, demonstrating a 4-mm density in the right lower quadrant Small, scattered amounts of free intraperitoneal fluid are observed without a dilated appendix and mild inflammatory changes.

On initial physical examination, the patient was awake, active, and consolable with a non-toxic appearance. Vital signs were as follows: heart rate: 142 beats per minute; respiratory rate: 42 breaths per minute; blood pressure: 106/60 mm Hg; and temperature: 40.4°C. She had dry mucous membranes and peripheral extremities were cool to touch. All other initial examination findings were benign, including rectal examination and stool guaiac testing, which were also unremarkable. Pertinent laboratory testing revealed leukopenia with a left shift and elevated C-reactive protein (CRP) levels. Other routine test results, including urinalysis, lactic acid, and serum electrolytes, were within normal reference ranges. An initial abdominal radiograph (Figure 1) revealed non-obstructive bowel distention without any evidence of discrete masses or abnormal calcifications. The patient was admitted and started on intravenous (IV) ceftriaxone after receiving appropriate fluid therapy. However, the patient’s fever persisted overnight despite therapeutic and supportive treatment with antipyretics. Moreover, the patient’s clinical hydration status did not improve after two boluses of IV fluids and maintenance fluids. On repeat examination, the patient developed findings that were suspicious of an acute abdomen, in addition to tachycardia and tachypnea, increased abdominal distension, hypoactive bowel sounds, guarding, and generalized tenderness. Following a shared decision-making discussion with the parents regarding the risks and benefits of further radiation exposure, a repeat abdominal radiograph (Figure 2) revealed a 9-cm soft tissue mass, prompting further evaluation with abdominal ultrasonography, which revealed a soft tissue mass with central fluid densities located above the umbilicus. Due to its indiscernible etiology, a computed tomography (CT) was performed, which revealed findings highly suspicious of a ruptured appendix with an appendicular mass (Figure 3). A diagnosis of peritonitis secondary to a ruptured appendicular mass was given. Immediately, IV antibiotic coverage was further broadened and the patient was transferred to the critical care unit for more intensive management. However, the option for emergent surgery was made available if the patient’s clinical status deteriorated. During the following few days, the patient’s septic picture improved. The parents elected to perform an elective appendectomy after resolution.

## Discussion

Although acute appendicitis is uncommon in infants and children under the age of four, it remains one of the common causes of abdominal pain in children with incidences of 3.6/10,000–1.1/10,000 in preschoolers and 18.6/10,000–6.8/10,000 in children aged five to nine years [2]. These incidences generally increase with age and peak during teenage years, subsequently declining into adult years. The exact pathophysiology of AA remains unclear and is likely a multifactorial etiology. Luminal obstruction is almost always evident and lymphoid hyperplasia, rather than a fecolith, is the usual culprit among toddlers. Because the incidences of AA are lower in western countries, there have been discussions pertaining to whether this observation is due to increased recognition or due to environmental factors, such as diet and hygiene [5]. However, environmental factors are difficult to discern, particularly in the very young whose exposure history is limited, similar to our patient.

The clinical presentations of AA in young children are often subtle, with non-specific symptoms, and obtaining a reliable history is more difficult to acquire from the young historian. While up to a third of children may present with the classical constellation of symptoms associated with AA, preverbal children and toddlers often present atypically. In an 18-year review of AA in young children, the most common symptoms were found to be vomiting (90%), fever (60%), abdominal distention (52%), diarrhea (46%), and lethargy (40%), which are summarized in Table 1 [2]. These non-specific symptoms are often short-lived and intermittently asymptomatic, making the diagnosis difficult. Similar to the abovementioned case, our patient’s presentation was initially benign and rapidly deteriorated with non-specific symptoms.

**Table 1:** Summary of commonly observed findings in acute appendicitis among young children (

Signs & Symptoms
Vomiting
Pain
Fever
Diarrhea
Cough
Rhinitis
Respiratory distress
Decreased right hip mobility/limping
Abdominal distension/rigidity
Palpable mass
Irritability
Lethargy

Delays in diagnosis increase the risk of dire complications, of which the most common complications include perforation and obstruction (80%-90%) [2,6]. These delays have been associated with increases in morbidity and mortality, which particularly increase in the presence of an intra-abdominal abscess or peritonitis, similar to our patient. Some have reported an estimated mean diagnostic time interval (from symptom onset to diagnosis) is around 3–4 days [2,6]. Furthermore, the diagnostic overlap with other common conditions increases the risk of misdiagnosis of AA. The differential in children is quite extensive and include several common etiologies, such as acute gastroenteritis, constipation, Henoch-Schönlein purpura, intussusception, lower lobar pneumonia, Meckel’s diverticulum, mesenteric lymphadenitis, urinary tract infection, and pyelonephritis. Nevertheless, misdiagnosis remains a common cause of medical malpractice in pediatric emergency care. Thus, a high index of suspicion along with prompt intervention is required [2-4].

The utilization of CT imaging is highly sensitive for the diagnosis of AA and has been increasingly used for diagnosis in children. However, exposure to radiation remains a primary concern and often steers clinicians into utilizing ultrasound imaging as the first-line choice in these young patients due to its specificity. Still, the sensitivity of abdominal ultrasound in the diagnosis of AA in young children is observed to be at 50% and is highly dependent on the technical skills of the operator [7]. Some have suggested modifying those diagnostic accuracies by establishing some standardized diagnostic criteria. Several diagnostic studies have been proposed and are summarized in Table 2. Recently, Nishizawa et al. conducted a retrospective study of 328 children to determine which children may be considered for a CT following a non-diagnostic ultrasound and found that CT is most beneficial if they met certain criteria [8]. Future studies will shed more light on this topic.

**Table 2:** Summary of commonly utilized diagnostic methods for acute appendicitis in young children CBC, complete blood count; CRP, C-reactive protein; N/L, Neutrophils to lymphocytes; CT, Computed Tomography; n/a, not available.

Commonly utilized diagnostic methods for acute appendicitis in young children	
Method	Comments
CBC	SN 60–87%, SP 53–100%.
CRP	SN 43%–92%, SP 33%–95%; may be more sensitive than CBC in diagnosing appendicular perforation and abscess formation.
N/L ratio	Maybe a more sensitive indicator than CBC.
Urine analysis	Better at discriminating simple and perforated appendicitis
Radiography	More specific for the diagnosis of AA due to fecolith.
Ultrasonography	SN 80%–92%, SP 86%–98%
CT	SN 87%–100%, SP 83%–100%; may reduce negative appendectomy rate.
Barium enema	n/a
Radioactive leukocyte scan	n/a
Diagnostic laparoscopy	n/a

## Conclusions

Acute appendicitis is a serious and potentially life-threatening condition that is not commonly observed in young children. The challenge remains in unreliable examinations, the usefulness of laboratory testing, the relevance of clinical scoring systems, and the ethics of diagnostic management. Atypical presentation is common in children under the age of four. Moreover, the diagnostic overlap with other common conditions increases the risk of misdiagnosis. Concerns with radiation exposure may delay the diagnosis and increase the risk of perforation and peritonitis. Clinicians should have a high index of suspicion for AA, particularly in young children, as this condition is commonly missed on initial presentation.

## References

[1] Schwartz KL, Gilad E, Sigalet D, Yu W, Wong AL (2011). Neonatal acute appendicitis: a proposed algorithm for timely diagnosis. J Pediatr Surg.

[2] Marzuillo P, Germani C, Krauss BS, Barbi E (2015). Appendicitis in children less than five years old: a challenge for the general practitioner. World J Clin Pediatr.

[3] Almaramhy HH (2017). Acute appendicitis in young children less than 5 years: review article. Ital J Pediatr.

[4] Armstrong J, Merritt N, Jones S, Scott L, Bütter A (2014). Non-operative management of early, acute appendicitis in children: is it safe and effective?. J Pediatr Surg.

[5] Papadopoulos AA, Polymeros D, Kateri M, Tzathas C, Koutras M, Ladas SD (2008). Dramatic decline of acute appendicitis in Greece over 30 years: index of improvement of socioeconomic conditions or diagnostic aids?. Dig Dis.

[6] Chang YT, Lin JY, Huang YS (2006). Appendicitis in children younger than 3 years of age: an 18-year experience. Kaohsiung J Med Sci.

[7] Choi JY, Ryoo E, Jo JH, Hann T, Kim SM (2016). Risk factors of delayed diagnosis of acute appendicitis in children: for early detection of acute appendicitis. Korean J Pediatr.

[8] Nishizawa T, Maeda S, Goldman RD, Hayashi H (2018). Predicting need for additional CT scan in children with a non-diagnostic ultrasound for appendicitis in the emergency department. Am J Emerg Med.

